# Supplementary material for: Electrochromic semiconductors as colorimetric SERS substrates with high reproducibility and renewability
Source: Nat Commun. 2019 Feb 8;10:678. doi: 10.1038/s41467-019-08656-6 (PMC6368540; doi:10.1038/s41467-019-08656-6)
Supplement: Supplementary file 1 — Supplementary Information [file 41467_2019_8656_MOESM1_ESM.pdf]

Supplementary Information

Electrochromic Semiconductors as Colorimetric SERS Substrates with High Reproducibility and Renewability

Cong et al.

Supplementary Figures

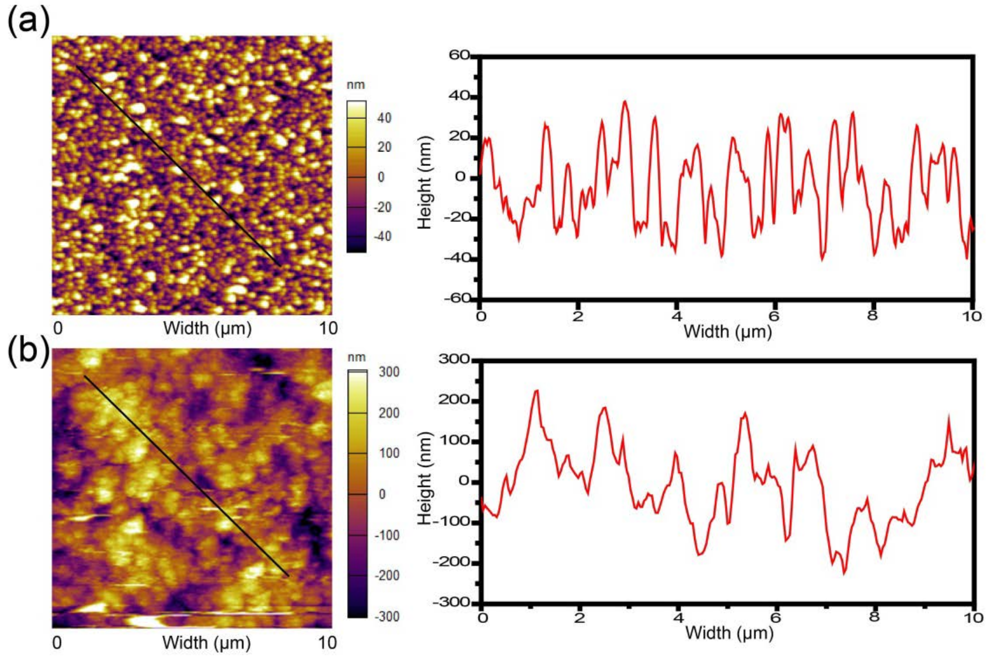

Supplementary Figure 1. AFM measurements show low variation in the surface roughness for (a) the sputtered tungsten oxide film, and relative large surface roughness for (b) the tungsten oxide prepared by drop-casting method.

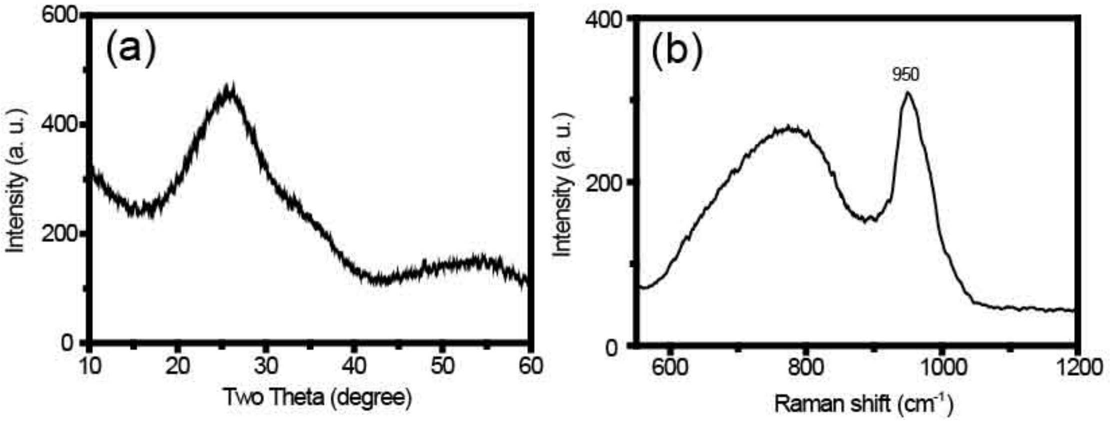

Supplementary Figure 2. (a) XRD pattern and (b) Raman spectrum for the tungsten oxide film, implying the amorphous nature.

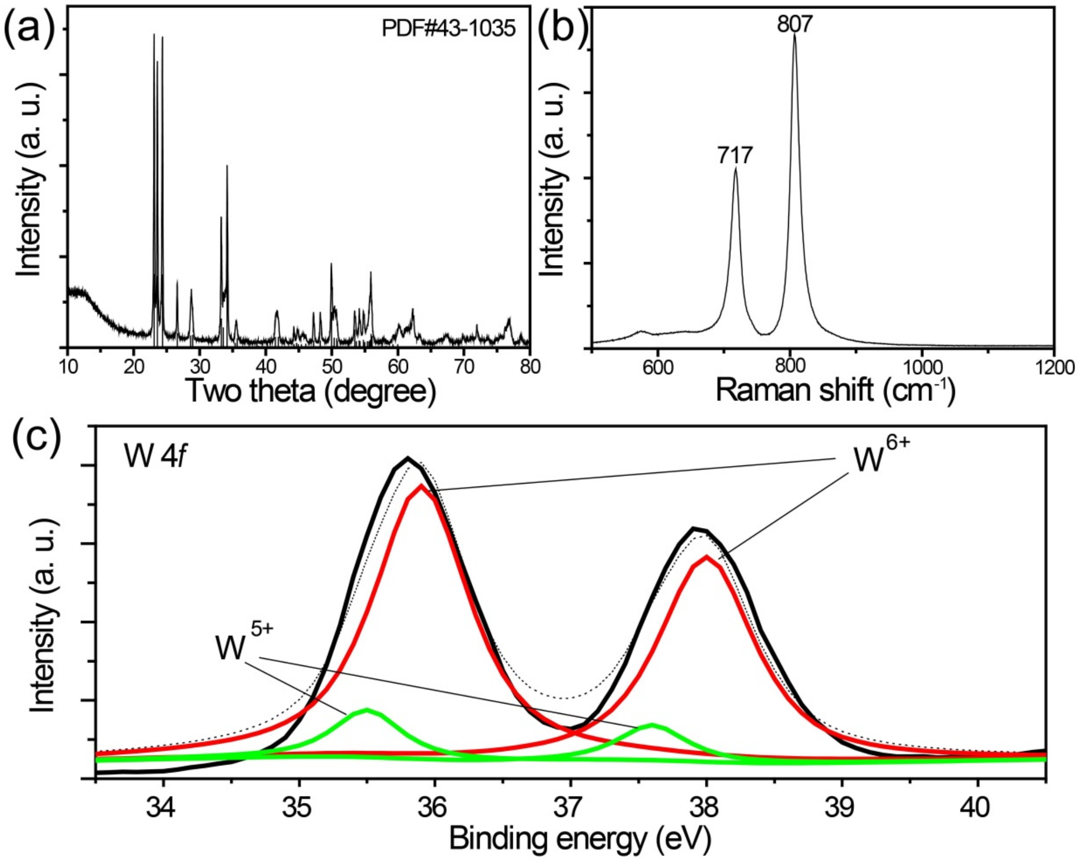

Supplementary Figure 3. (a) XRD pattern, (b) Raman spectrum, and (c) XPS spectrum of the commercially available crystalline tungsten oxide powder (monoclinic). The XRD pattern of the commercial tungsten oxide powder corresponds to the monoclinic structure of WO_3 (lattice parameters: a = 7.297 Å, b = 7.539 Å, c = 7.688 Å, β = 90.91°, JCPDS no: 43-1035), showing typical crystalline characteristics with a series of prominent diffraction peaks at 2θ angles of 20-80°. Raman bands occur at 717 (O-W-O vibration) and 807 cm^{-1} (W-O-W stretching), which further confirm the monoclinic structure of the commercial tungsten oxide powder. XPS analysis suggests the coexistence of W in its +5 and +6 oxidation states in the commercial tungsten oxide powder, although the ratio of W^{5+}/W^{6+} is quite small (10.7 : 89.3).

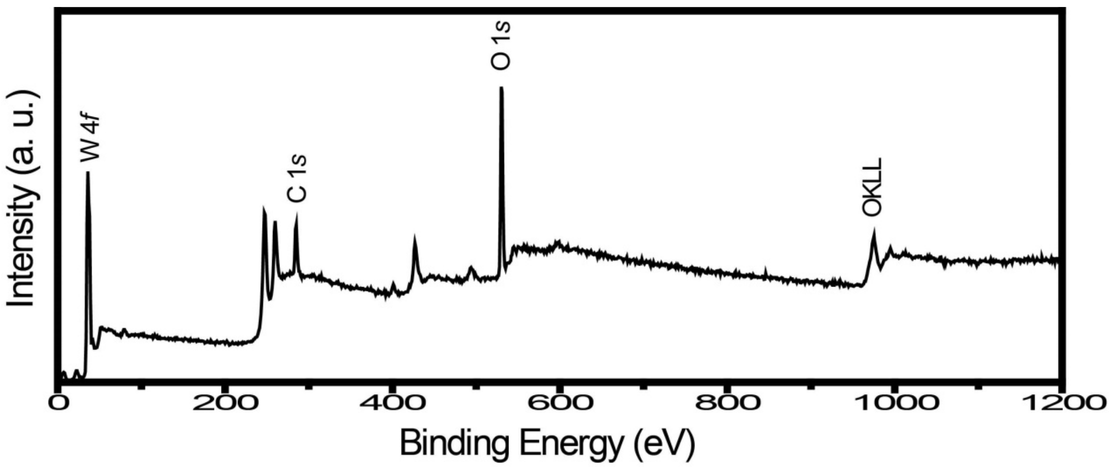

Supplementary Figure 4. The XPS survey spectra show that the as-deposited film mainly consists of W and O elements.

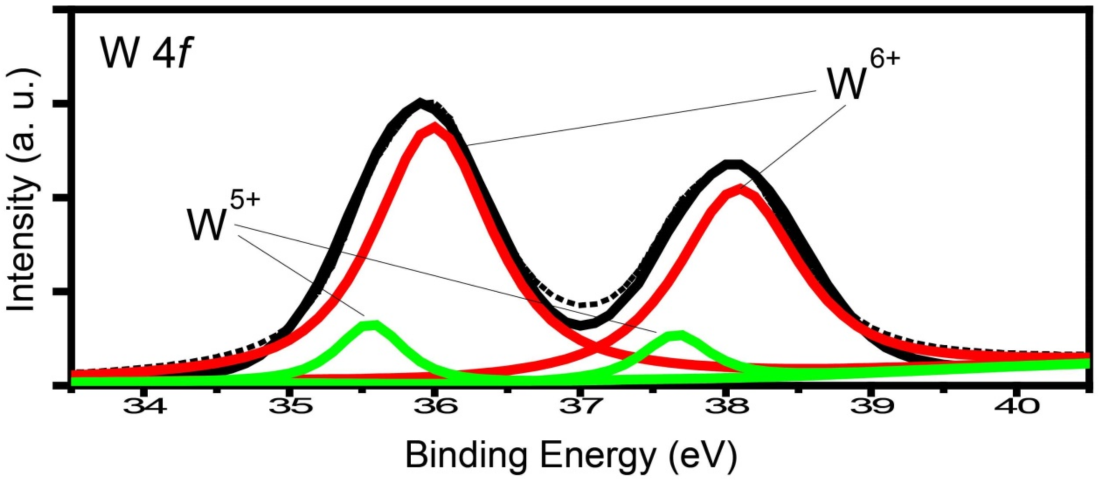

Supplementary Figure 5. W 4*f* core levels of the XPS spectra for pristine tungsten oxide film.

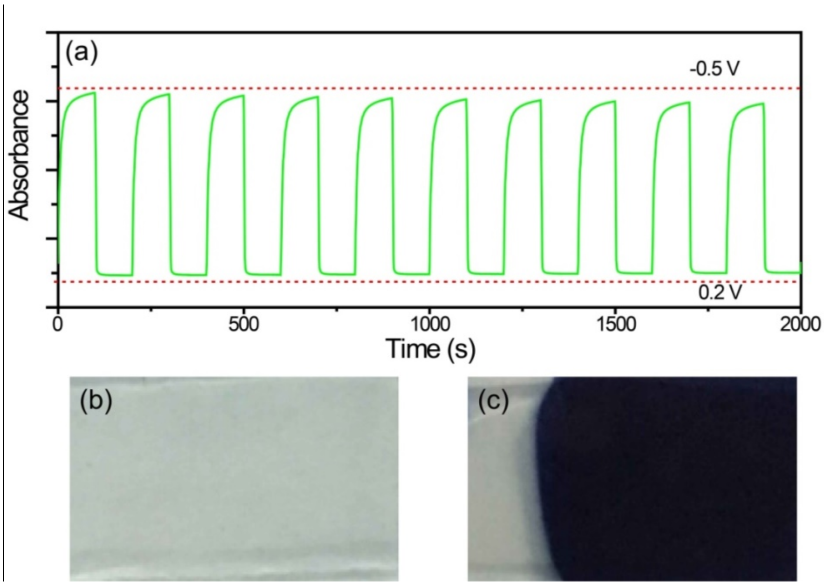

Supplementary Figure 6. (a) Electrochromic switching, optical absorbance monitored at 532 nm for the sputtered tungsten oxide film, with alternating bias potentials switching between -0.5 and 0.2 V in 1 M $AlCl_3$ aqueous solution. High-resolution photos of the tungsten oxide film in its bleached (b) and colored (c) states.

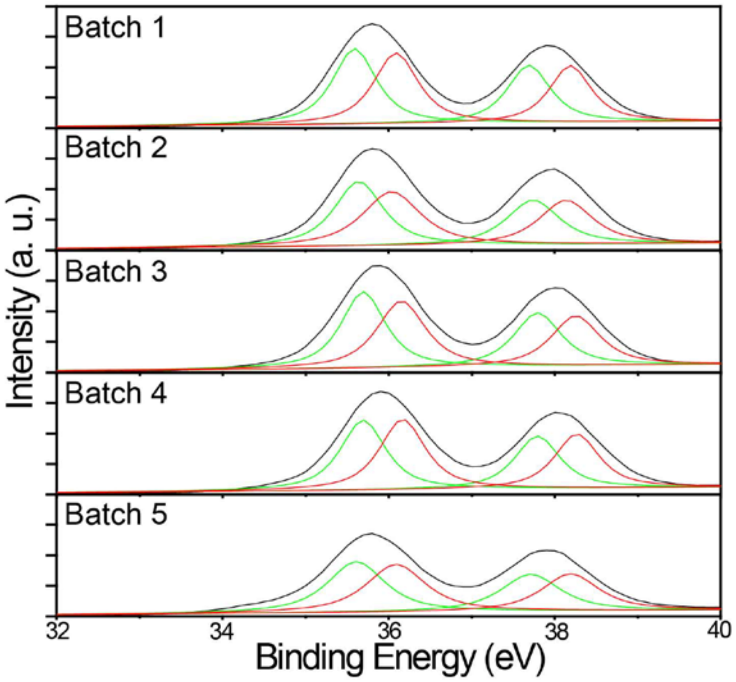

Supplementary Figure 7. W 4*f* core levels of the XPS spectra for the Al-intercalated tungsten oxide films from five batches, prepared using the same experimental conditions.

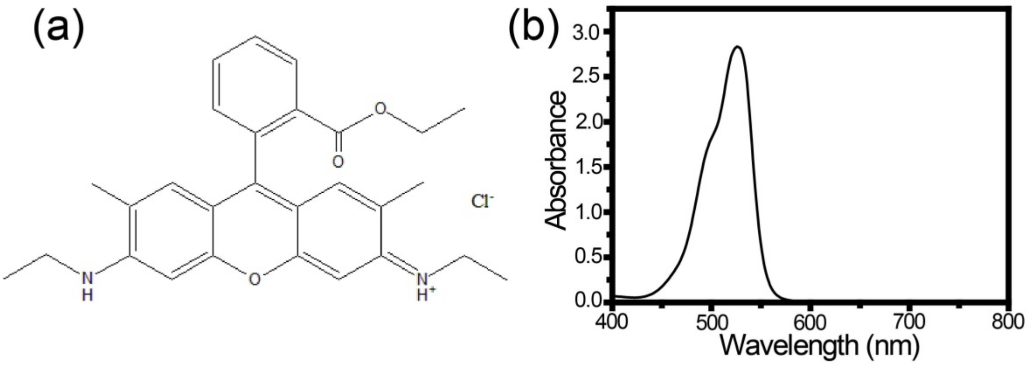

Supplementary Figure 8. (a) Molecular structure and (b) absorption spectrum of R6G.

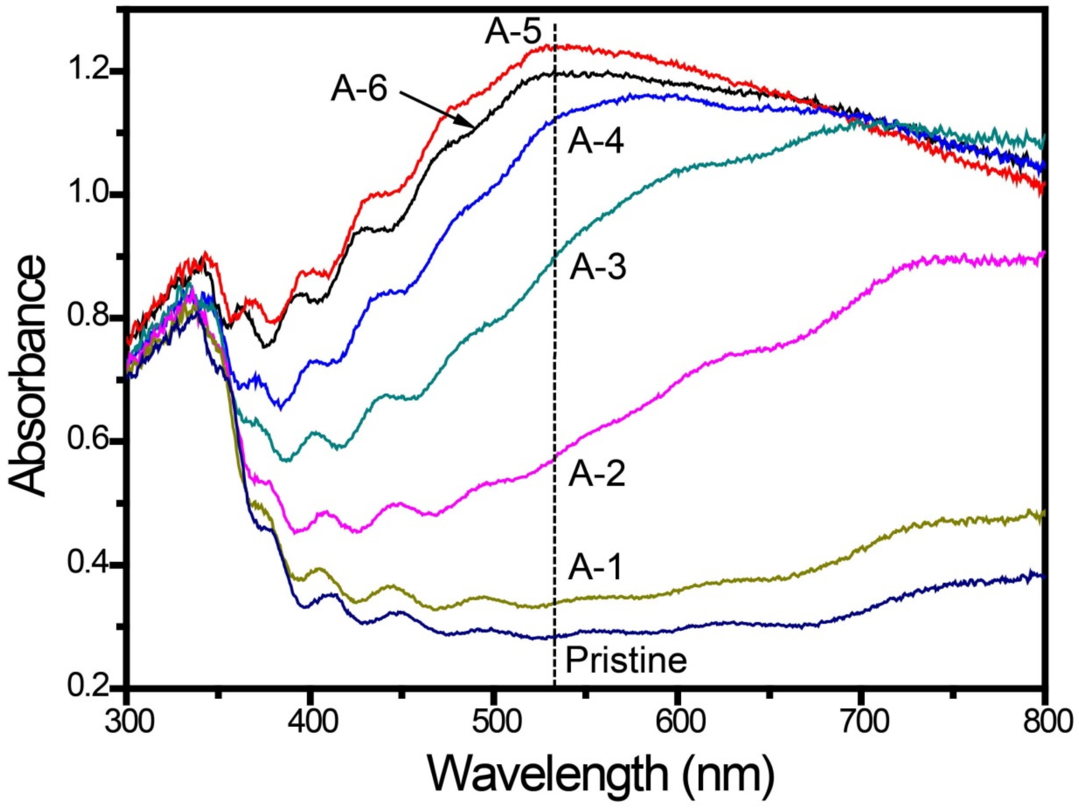

Supplementary Figure 9. The absorbance of the pristine and colored tungsten oxide films monitored in the 300-800 nm wavelength range. Colored films are obtained via Al-intercalation at various negative potentials, -0.1, -0.2, -0.3, -0.4, -0.5 and -0.6 V, denoting as Al-1, Al-2, Al-3, Al-4, Al-5 and Al-6, respectively.

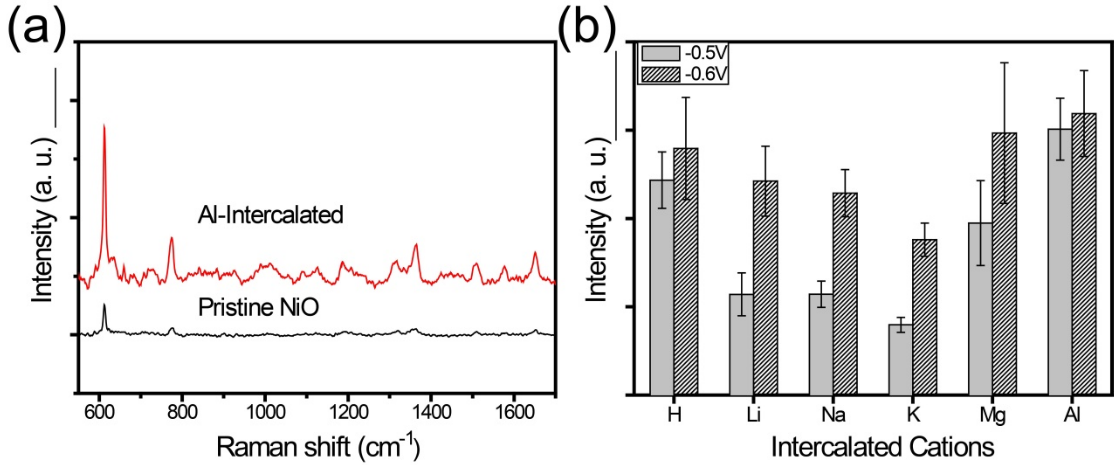

Supplementary Figure 10. (a) Raman spectra for R6G (10^{-4} M) on pristine NiO and Al-intercalated NiO film (scale bar: 500 cps). (b) The Raman intensities of vibrational band at 612 cm^{-1} of R6G on the substrates intercalated with varied cations. Cation-intercalation is conducted via the Chronoamperogram method, at constant potential of -0.5 and -0.6 V for 180 s, in 1 M $AlCl_3$ aqueous analyte solution, respectively (scale bar: 500 cps). Error bars represent means ± SD of the SERS intensity.

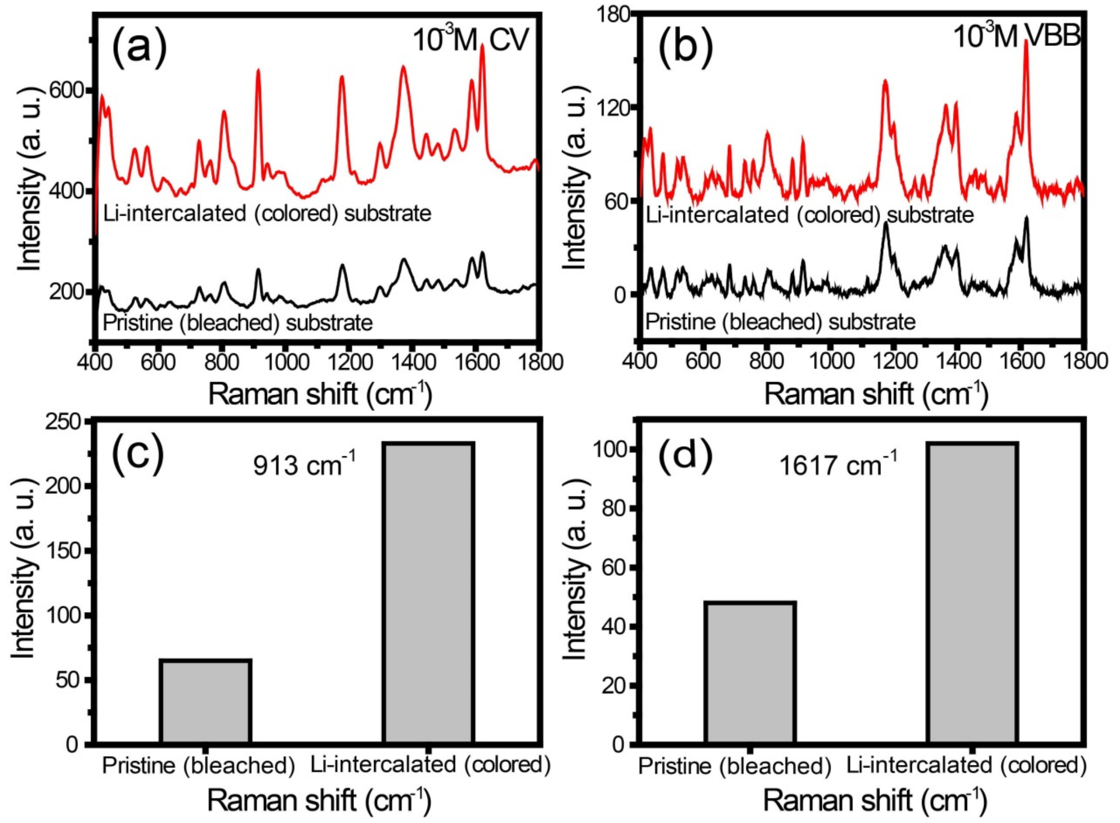

Supplementary Figure 11. SERS spectra of (a) crystal violet (10^{-3} M) and (b) Victoria blue B (10^{-3} M) on the pristine (bleached) and Li-intercalated tungsten (colored) oxide films under 532 nm laser excitation. A comparison of SERS activity between the pristine (bleached) and Li-intercalated (colored) tungsten oxide film under 532 nm laser excitation, for (c) vibrational band at 913 cm^{-1} of CV and (d) vibrational band at 1617 cm^{-1} of VBB. Cation intercalation is conducted in 1 M aqueous LiCl analyte solution via chronoamperometry at constant potential of -0.5 V for 180 s.

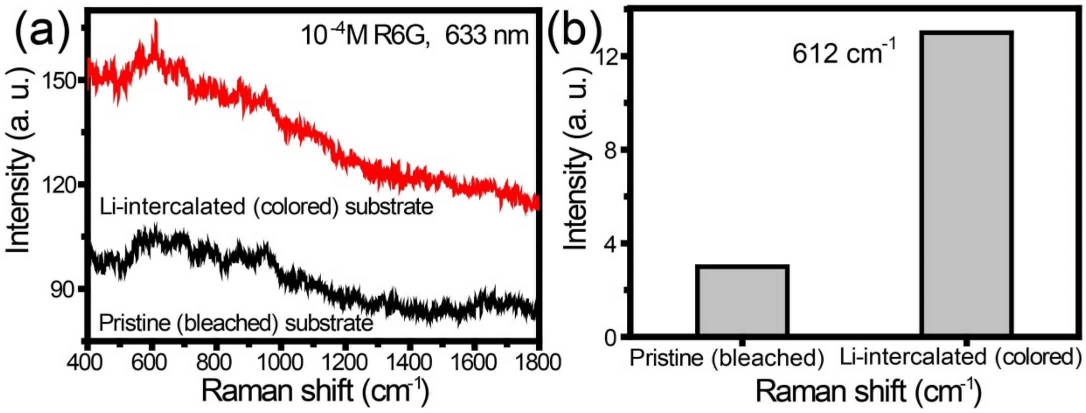

Supplementary Figure 12. (a) SERS spectra of R6G (10^{-4} M) on the pristine (bleached) and Li-intercalated tungsten oxide films (colored) under 633 nm laser excitation. A comparison of SERS activity between the pristine (bleached) and Li-intercalated (colored) tungsten oxide film under 633 nm laser excitation for vibrational band at 612 cm^{-1} of R6G. Cation intercalation is conducted in 1 M aqueous LiCl analyte solution via chronoamperometry at constant potential of -0.5 V for 180 s.

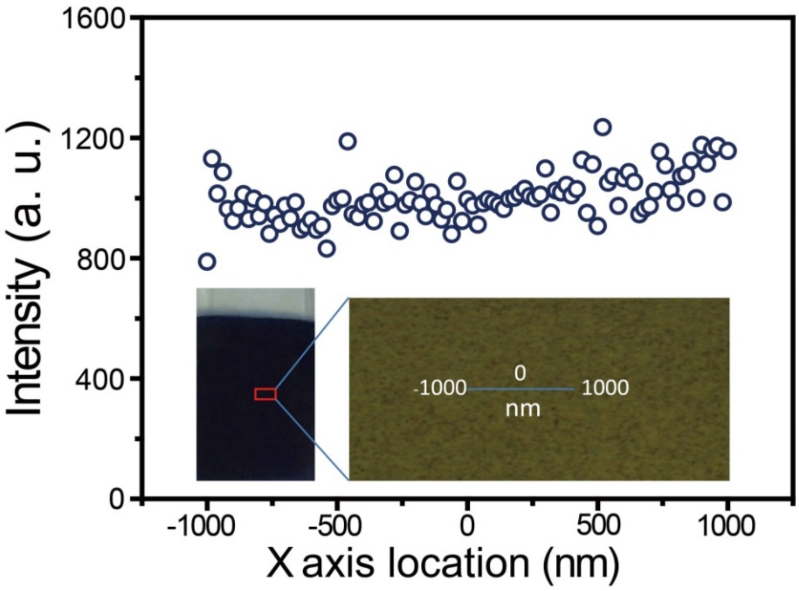

Supplementary Figure 13. Variation of SERS intensity of 612 cm^{-1} vibrational band across a randomly selected area on the surface of single Al-5 substrate. Inset photo marks the selected area with its magnified morphology obtained under microscope of the Raman instrument.

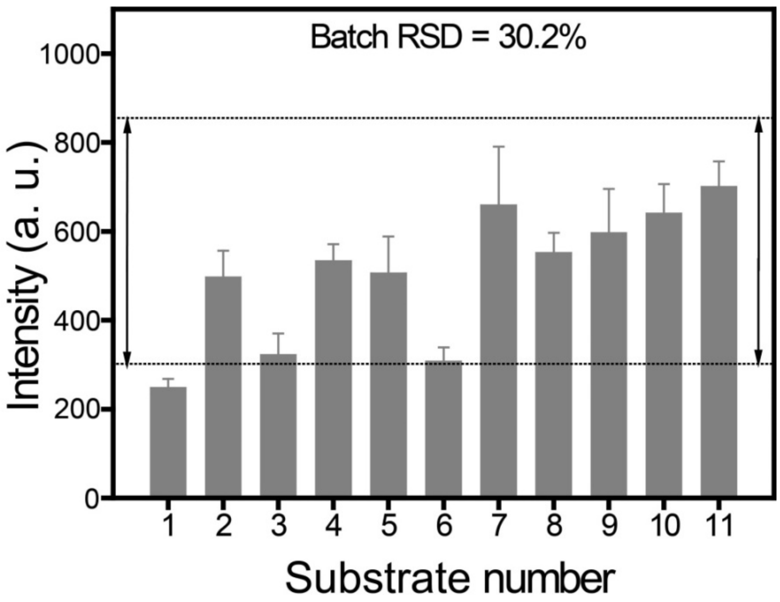

Supplementary Figure 14. Mean intensities of the vibrational band (612 cm^{-1}) of R6G on randomly selected tungsten oxide substrates pretreated by H_2-thermalreduction under the same condition. The RSD value among batches is calculated to be 30.2%. Error bars represent means ± SD of the SERS intensity.

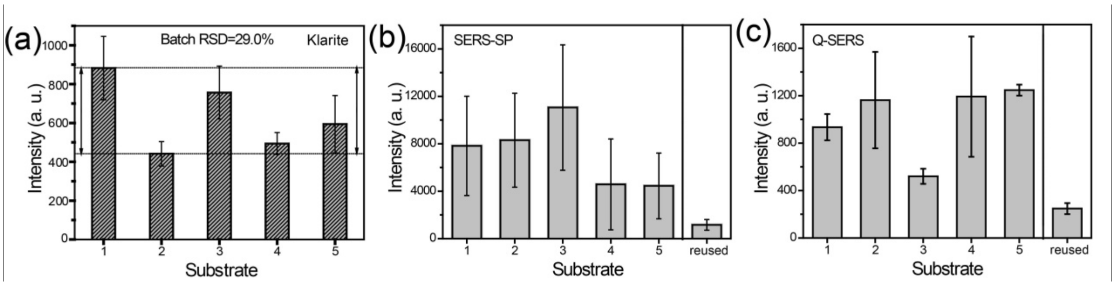

Supplementary Figure 15. (a) Average intensities of the vibrational band (612 cm^{-1}) of R6G (10^{-4} M) under 532 nm laser excitation obtained from three types of commercially available substrates, (a) Klarite, (b) SERS-SP and (b) Q-SERS. Signal intensities on five pieces of SERS substrate in different batches are acquired to calculate the batch-to-batch RSD values, which is 29.0%, 38.4% and 29.6% for Klarite, SERS-SP and Q-SERS, respectively. It is also found that the one-time use of recycled substrates causes dramatical decrease in signal intensity for commercial substrates (SERS-SP and Q-SERS). Note that all samples for SERS measurement are prepared by casting 20 μl of 10^{-4} M R6G in ethanol onto the surface of SERS substrates (area: 4×4 mm^2), allowing the solvent to evaporate. To ensure equal amount of analyte loading, the generated droplets have almost the same volume when using the microburets. Error bars represent means ± SD of the SERS intensity.

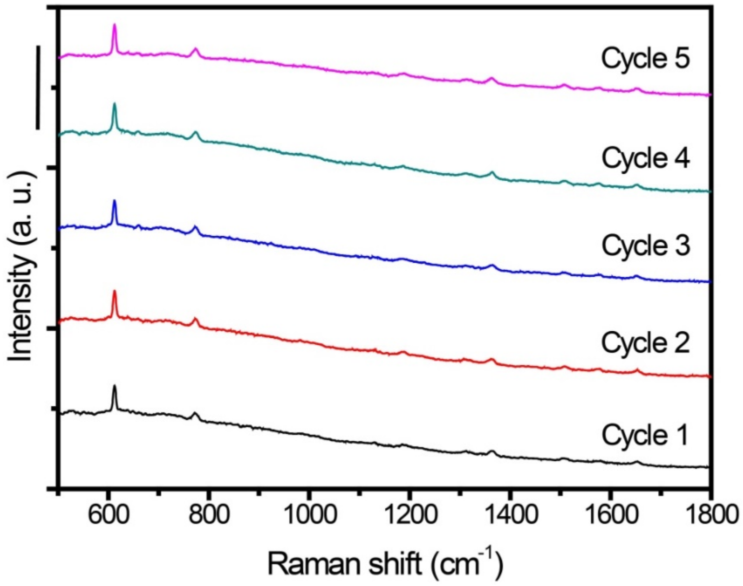

Supplementary Figure 16. The collected SERS spectra from single SERS substrates (Al-5) after successively coloring-decoloring cycles for regeneration, which are compared with that obtained from the freshly-prepared substrate (denoted as Cycle 1) (scale bar: 2000 cps).

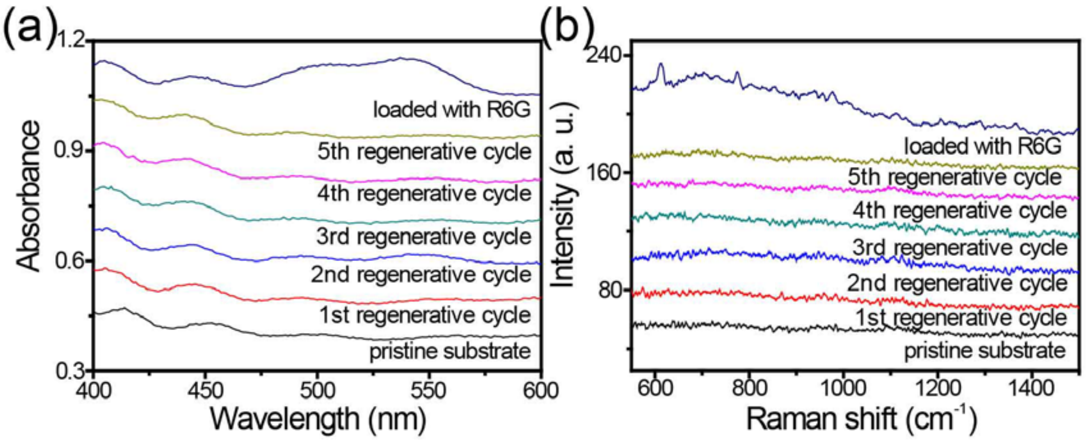

Supplementary Figure 17. (a) UV-Visible absorption spectra and (b) Raman spectra at 532 nm laser excitation of a series of substrates including the pristine tungsten oxide film, the tungsten oxide film loaded with R6G analytes (10^{-6} M) and the regenerated tungsten oxide films with different regenerative cycles by means of electrochromic treatments.

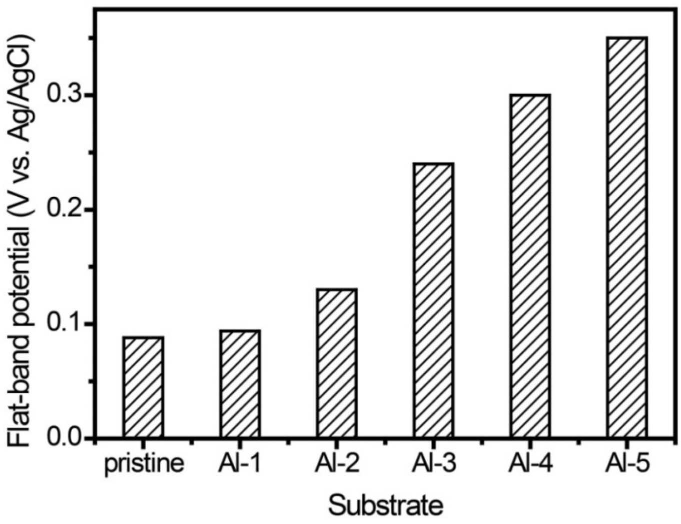

Supplementary Figure 18. Flat-band potential (with respect to Ag/AgCl electrode) obtained from the Mott-Schottky plots for pristine tungsten oxide and Al-intercalated films, sorted with decreasing potentials for Al-intercalation.

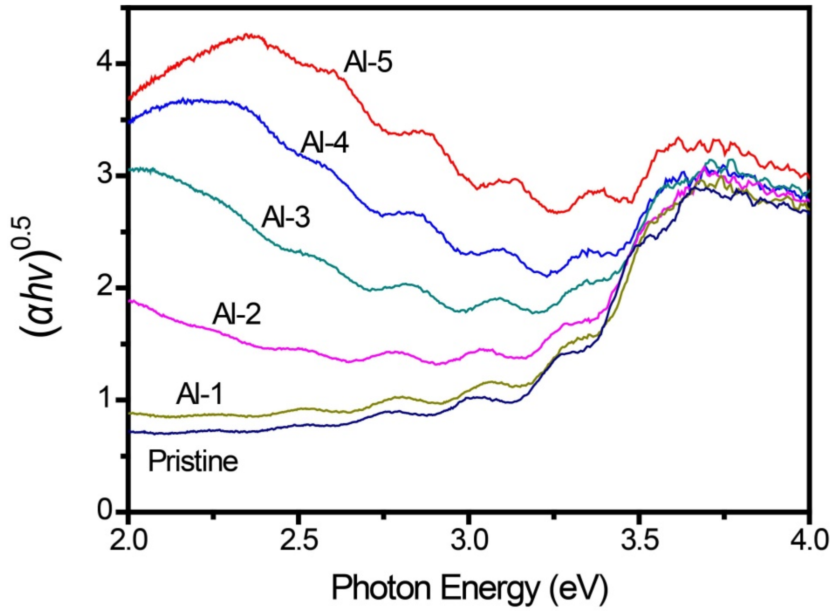

Supplementary Figure 19. UV-Vis diffuse reflectance spectra recorded in Kubelka-Munk unit, giving band-gap energy values by the intersection of the line tangent to the plotted curve inflection point with the horizontal axis, for pristine tungsten oxide and Al-intercalated films.

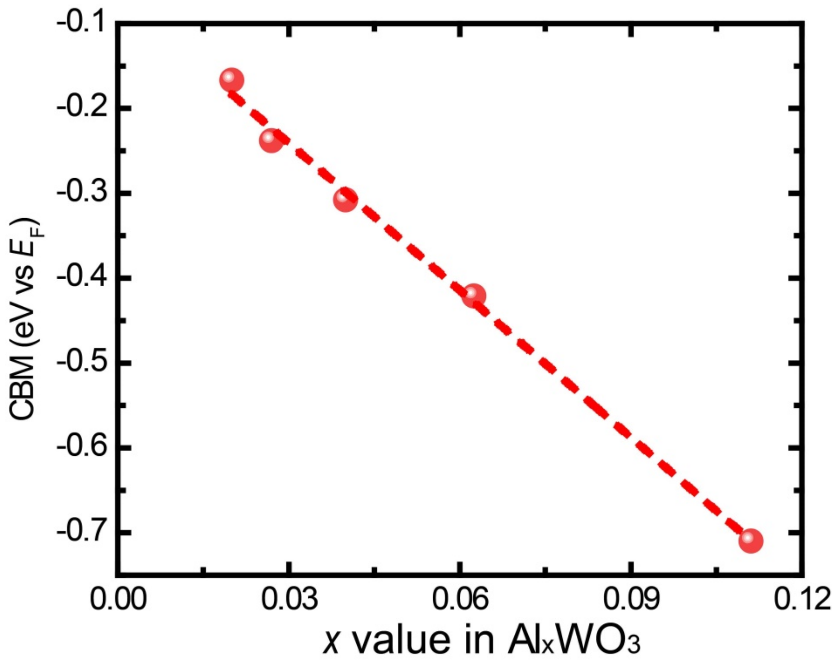

Supplementary Figure 20. Calculated conduction band minimum (CBM) with respect to Fermi level (E_F), showing a linear down-shifting as x value in Al_xWO_3 increases from 0 to 0.12.

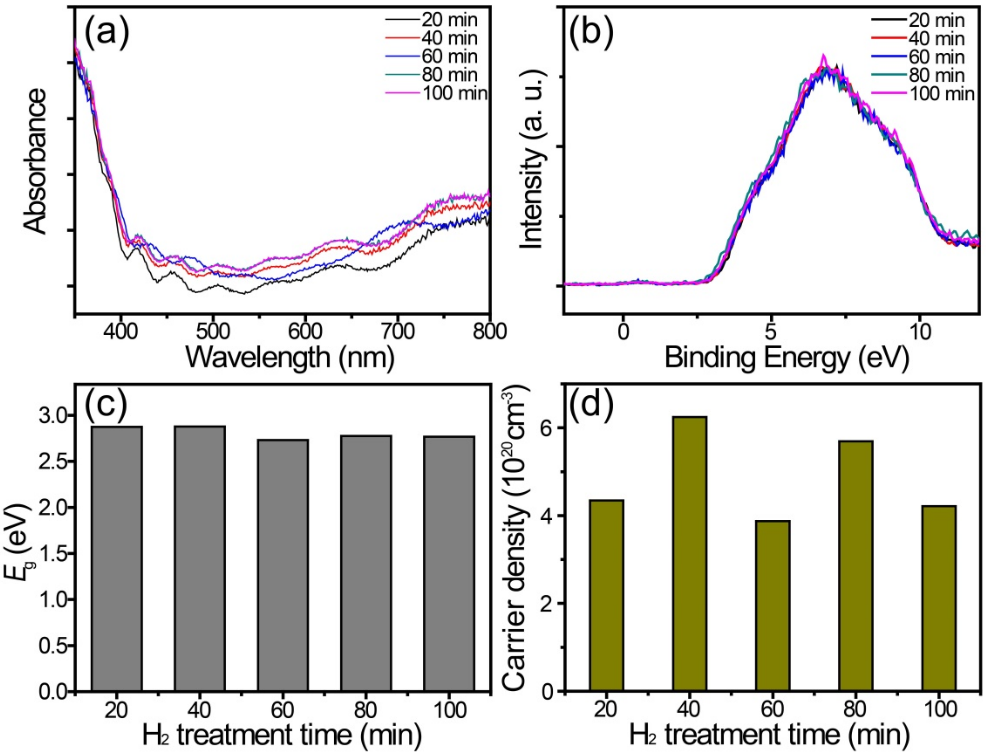

Supplementary Figure 21. (a) UV-Vis absorbance spectra, (b) XPS valence band spectra, (c) band-gap energy values obtained from UV-Vis absorbance spectra, and (d) carrier density calculated from Mott-Schottky plots, for H_2-treated tungsten oxide substrates at an elaborately controlled H_2 flow at 350 ^{o}C with treating time from 20 to 100 min.

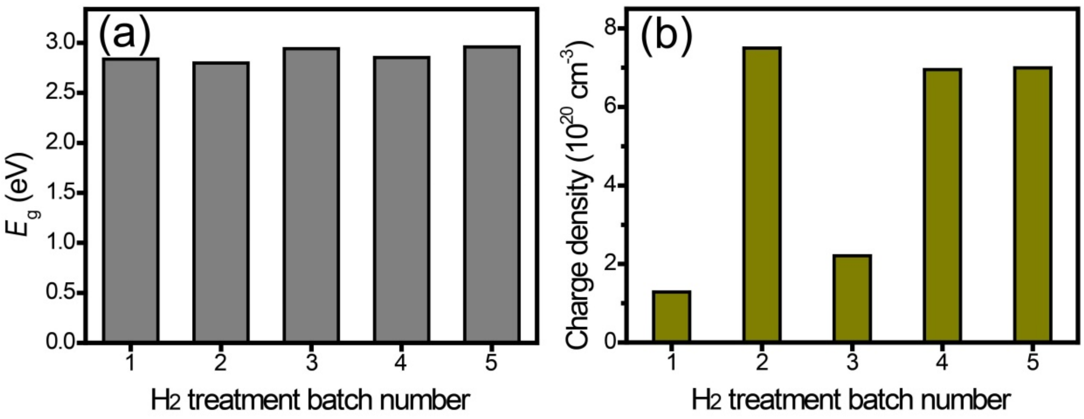

Supplementary Figure 22. (a) Band-gap energy values obtained from UV-Vis absorbance spectra, and (b) carrier density calculated from Mott-Schottky plots, for a batch of H_2-treated tungsten oxide substrates at an elaborately controlled H_2 flow at 350 ^{o}C with treating time of 30 min.

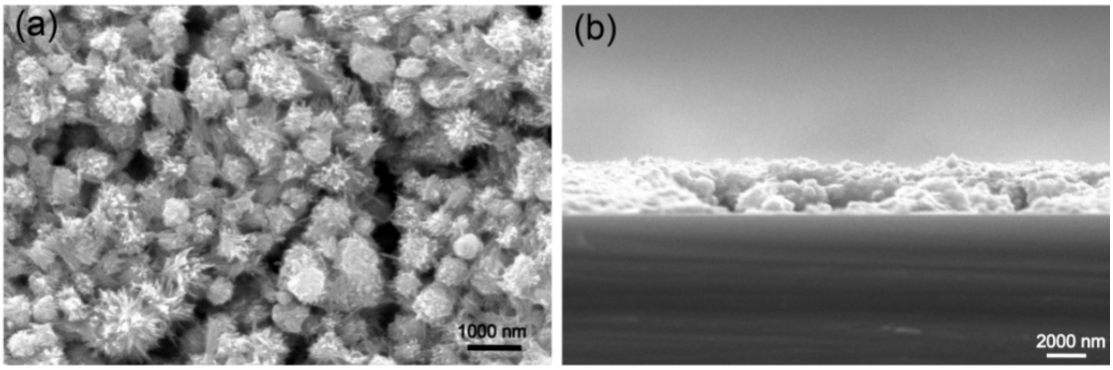

Supplementary Figure 23. (a) Top-view and (b) cross-sectional SEM images of the tungsten oxide film prepared by drop-casting method.

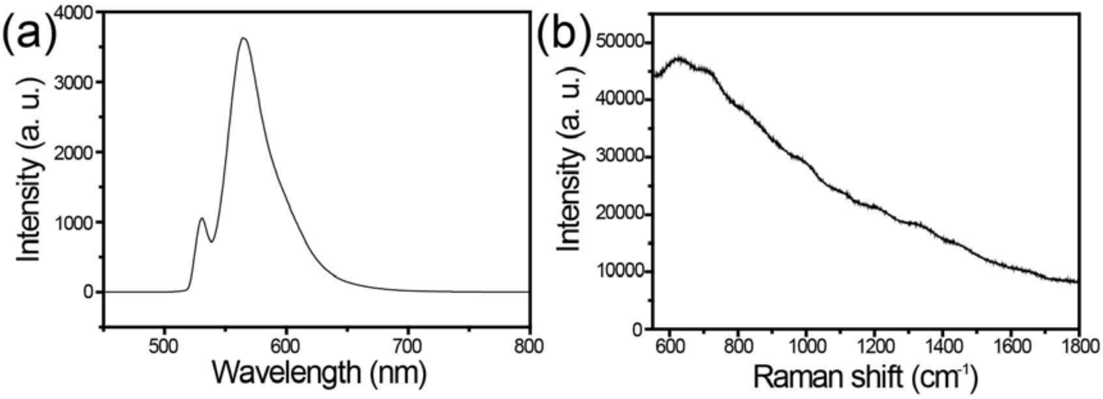

Supplementary Figure 24. (a) Fluorescence spectrum of R6G with the excitation wavelength of 532 nm. (b) Raman spectra of R6G (10^{-4} M) on the electrochromic tungsten oxide film and bare SiO_2/Si substrate under 532 nm laser excitation, without baseline correction. The electrochromic tungsten oxide film is prepared in 1 M aqueous $AlCl_3$ analyte solution via chronoamperometry at constant potential of -0.5 V for 180 s.

Supplementary Table

Supplementary Table 1. Band structure information of pristine and Al-intercalated films

	Unit	Pristine	Al-1	Al-2	Al-3	Al-4	Al-5
**V_{fb} vs. Ag/AgCl**	V	0.088	0.094	0.13	0.24	0.3	0.35
**E_F**	eV	-0.285	-0.291	-0.327	-0.437	-0.497	-0.547
**VB**	eV	3.175	3.169	3.133	3.023	2.963	2.913
**E_g**	eV	3.22	3.20	3.15	3.11	3.04	3.01
**CB**	eV	-0.045	-0.031	-0.017	-0.087	-0.077	-0.097
**Filling width**	eV	0.24	0.26	0.31	0.35	0.42	0.45

Note: The potential conversion relationship between NHE and Ag/AgCl reference electrode (saturated KCl solution) is: $E_{NHE} = E_{Ag/AgCl} + 0.197$ V[1]. Fermi level (E_F) can be determined using the relationship with flat-band potential (V_{fb}), $E_F = -eV_{fb}$, where e is the elementary charge[2]. With the relative location between VB and E_F given by XPS valence band spectra and E_g value obtained from UV-Vis absorption spectra, band structure of pristine and Al-intercalated tungsten oxide can be determined. Filling width indicates the energy gap between the Fermi level and the conduction band minimum.

Supplementary Methods

Characterizations.

The surface and cross-section morphologies of the film were measured using a FEI Quanta 400 FEG field emission scanning electron microscope (SEM). XRD patterns of the samples were recorded on a Bruker AXS D8 Advance X-ray diffractometer with a Cu Kα radiation target (40 V, 40 A). UV-Vis absorption measurement was carried out on a UV-Vis spectrophotometer (V660, JASCO) over a wavelength range of 300-800 nm. XPS measurements were performed on a PHI Quantera XPS Scanning Microprobe spectrometer with Al Kα ($h\nu$ = 1486.6 eV) as the X-ray

radiation source, which had been carefully calibrated on valence band.

Renewing of the SERS substrates.

Electrochromic renewing of tungsten oxide SERS substrates. Typically, following extensive washing with distilled water and/or ethanol, the adsorbed R6G molecules are further detached from the tungsten oxide substrate after SERS measurement in an aqueous electrolyte solution by applying a reverse bias of 0.2 V for 180 s. Subsequently, the electrochromic-treated substrate is re-colored to the same degree as the pristine one under a voltage of -0.5 V after again washing with distilled water and/or ethanol. Then, the reactivated substrate could be re-used for the next SERS measurement after re-loading of R6G analyte. This procedure can be repeated many times as necessary. Notably, the R6G molecules are believed to interact with our electrochromic substrates by both physisorption and chemisorption, since some part of R6G are easily detached when immersed into ethanol while some part of R6G are in no way washed away by ethanol or water. Expectedly, the chemisorption of R6G occurs at disordered regions, particularly defects on tungsten oxide substrate (i.e. oxygen vacancies, W^{5+}) by electrostatic interaction.

Photocatalytic renewing of tungsten oxide SERS substrates. Typically, following extensive washing with distilled water and/or ethanol, the R6G-adsorbed substrates are illuminated for at least 2 hours by a simulated solar source equipped with a 300 W Xenon lamp (AM 1.5). Then, the reactivated substrate could be re-used for the next SERS measurement after again washing with distilled water and/or ethanol and re-loading of R6G analyte.

Calculation of the enhancement factor.

The enhancement factor EF was calculated according to the formula:

$$\mathrm{EF} = (I_{\mathrm{SERS}}/N_{\mathrm{SERS}})/(I_{\mathrm{bulk}}/N_{\mathrm{bulk}}) \quad (1)$$

$$N_{\mathrm{bulk}} = \rho h A_{\mathrm{Raman}} N_{\mathrm{A}}/M \quad (2)$$

$$N_{\mathrm{SERS}} = C V N_{\mathrm{A}} A_{\mathrm{Raman}}/A_{\mathrm{sub}} \quad (3)$$

N_{SERS} and N_{bulk} denote the number of R6G molecules that contribute to the signal intensity, enhanced and normal, respectively, while I_{SERS} and I_{bulk} denote the corresponding enhanced and normal Raman intensities (equation 1). As normal reference, the data for bulk R6G crystals on bare Si/SiO_2 wafer were acquired. h is the confocal depth of the laser beam, and on the basis of molecular weight (M) and density (ρ) of bulk R6G (1.15 g cm^{-3}), N_{bulk} is calculated by equation 2. For analyte molecules loaded with SERS-active substrates, N_{SERS} can be estimated by equation 3, assuming that the analyte was distributed uniformly on the surface of substrates. C is the molar concentration of the analyte solution, V is the volume of the droplet, N_A is Avogadro constant. A_{Raman} is the laser spot area (1 μm in diameter) of Raman scanning. 50 microliters of the droplet on the substrate was spread over the surface of the film (1.5 cm × 2.5 cm) spontaneously, from which the effective area of the substrate, A_{Sub}, can be obtained.

For the confocal depth (h) of the laser beam, recent references give different values for solid bulk crystalline R6G from 10, 13, 21 to 26 μm[4-7], which might be related to different pinhole sizes and objective lens of their used Raman instruments. To provide a more accurate estimate of the confocal depth in our system, a Raman intensity-depth profile of the 520.6 cm^{-1} band for a silicon wafer against the distance of it deviating from the ideally focused plane has been made in the light of the model assumed by Cai et al[6]. The pinhole size of 200 μm and a 50 × long working-length objective are used. Judging from the profile, the confocal depth (h) is determined to be 23.64 μm in our system.

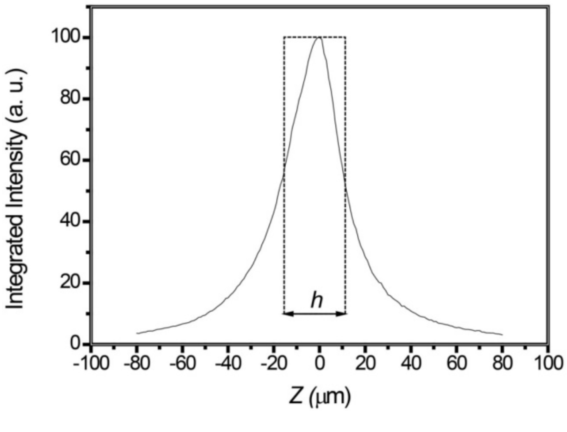

Charge-transfer theory.

To illustrate the contribution from electrons filled in CB to SERS enhancement, relationship between density of states (DOS) in CB and the intensity of a Raman transition is derived from the charge-transfer (CT) theory proposed by Lombardi et al for a molecule-metal system[8,9]. Based on the Herzberg-Teller theory regarding the vibronic coupling of the zero-order Born-Oppenhermer states, the contribution of CT to the molecule polarization tensor in the molecule-metal system can be estimated. Since the metal-like band structures of ion-intercalated tungsten oxide, we are mainly interested in the electronic behavior near the Fermi level, with the calculation especially concerning about the electronic states in partially filled CB derived according to the theory.

The intensity of a Raman transition can be expressed as a function of the polarizability tensor as the following,

$$I = [8\pi(\omega \pm \omega_{\mathrm{FI}})^4 I_{\mathrm{L}}/9c^4]\sum|\alpha_{\rho\sigma}|^2 \tag{4}$$

where I_{L} is the incident laser intensity at frequency ω, ω_{FI} is a molecular transition frequency between states I and F (presumably two different vibronic levels of the ground electronic state), σ, ρ are the scattered and incident polarization directions in space (X, Y, Z).

Following Lombardi et al, the general expression for the polarizability tensor may involve the sum of four terms：

$$\alpha_{\sigma\rho} = (A_f + A_k) + B + C \tag{5}$$

$$A_f = \left(\frac{2}{\hbar}\right) M_{\mathrm{SI}}^{\sigma 0} M_{\mathrm{SI}}^{\rho 0} \langle i|k\rangle\langle k|f\rangle \times \int_{\omega_{\mathrm{FI}}}^{\omega_{\mathrm{BI}}} \frac{(\omega_{\mathrm{SI}}+\omega_f)\rho_u(\omega_{\mathrm{SI}})d\omega_{\mathrm{SI}}}{(\omega_{\mathrm{SI}}+\omega_f)^2-\omega^2} \tag{6}$$

$$A_k = \left(\frac{2}{\hbar}\right) M_{\mathrm{SK}}^{\sigma 0} M_{\mathrm{SK}}^{\rho 0} \langle i|k\rangle\langle k|f\rangle \times \int_{\omega_{\mathrm{KA}}}^{\omega_{\mathrm{KF}}} \frac{(\omega_{\mathrm{KS}}+\omega_k)\rho_f(\omega_{\mathrm{KS}})d\omega_{\mathrm{KS}}}{(\omega_{\mathrm{KS}}+\omega_k)^2-\omega^2} \tag{7}$$

$$B = -\left(\frac{2}{\hbar^2}\right) \sum_{\mathrm{K}\neq\mathrm{I}} [M_{\mathrm{KI}}^{\sigma} M_{\mathrm{SI}}^{\rho 0} + M_{\mathrm{KI}}^{\rho} M_{\mathrm{SI}}^{\sigma 0}] \times \frac{h_{\mathrm{KS}}^0 \langle i|\mathrm{Q}|f\rangle}{(\omega_{\mathrm{KI}}^2-\omega)^2} \int_{\omega_{\mathrm{FI}}}^{\omega_{\mathrm{BI}}} \frac{(\omega_{\mathrm{KI}}\omega_{\mathrm{SI}}+\omega^2)\rho_u(\omega_{\mathrm{SI}})d\omega_{\mathrm{SI}}}{\omega_{\mathrm{SI}}^2-\omega^2} \tag{8}$$

$$C = -\left(\frac{2}{\hbar^2}\right) \sum_{\mathrm{K}\neq\mathrm{I}} [M_{\mathrm{SK}}^{\sigma 0} M_{\mathrm{KI}}^{\rho} + M_{\mathrm{SK}}^{\rho 0} M_{\mathrm{KI}}^{\sigma}] \times \frac{h_{\mathrm{IS}}^0 \langle i|\mathrm{Q}|f\rangle}{(\omega_{\mathrm{KI}}^2-\omega)^2} \int_{\omega_{\mathrm{KA}}}^{\omega_{\mathrm{KF}}} \frac{(\omega_{\mathrm{KI}}\omega_{\mathrm{KS}}+\omega^2)\rho_f(\omega_{\mathrm{KS}})d\omega_{\mathrm{KS}}}{\omega_{\mathrm{KS}}^2-\omega^2} \tag{9}$$

where K represents all the other states of the molecule, ω_k and ω_f are the

frequencies of a particular excited or ground state vibration, respectively. *h* is the coupling matrix element representing the degree to which a particular vibration *Q* can mix two states, *M* refers to the amplitudes of the corresponding transition moments between two states, and the terms with superscript *zero* are independent of energy. The conduction band ranges between ω_A and ω_B.

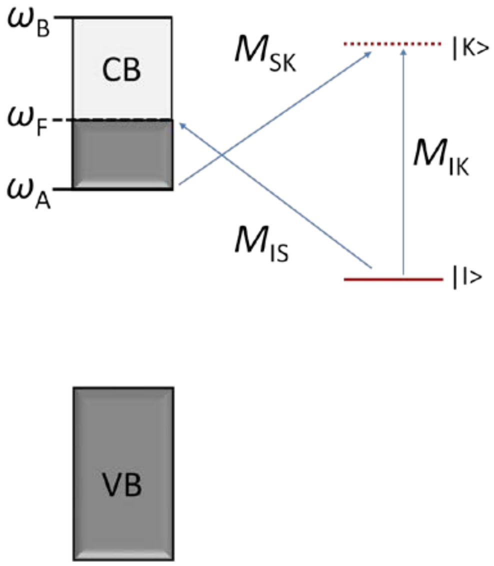

Term A_f represent resonant molecule-to-semiconductor charge transfer from the molecular ground state to one of the unfilled CB levels S, while A_k represents resonant semiconductor-to-molecule charge transfer from a filled state S in CB to an excited state K. Term *B* represents molecule-to-substrate charge transfer from the molecular ground state to one of the unfilled CB levels S, while the term *C* represents substrate-to-molecule charge transfer from one of the filled CB levels S to the excited state K.

Notably, ρ_u in A_f and *B* terms represents the density of unfilled CB states above the Fermi level for molecule-to-substrate transitions, while ρ_f in A_k and *C* terms refers to the density of filled states of CB for substrate-to-molecule transitions. To further simplify the mathematics, the function for the density of states near the Fermi level can be treated as constant for semiconductor with given partially-filled CB, according to the assumption representing the 0 K limit by Lombardi et al.[8].

$$\rho_u(\omega_{MI}) = \rho \quad \text{for} \ \ \omega_{FI} < \omega_{SI} < \omega_{BI} \tag{10}$$

$$\rho_f(\omega_{\mathrm{KM}}) = \rho \quad \text{for } \omega_{\mathrm{KA}}<\omega_{\mathrm{KS}}<\omega_{\mathrm{KF}} \tag{11}$$

By defining κ_{A} as the coefficient of the integrals in A_f and A_k, $\kappa_{\mathrm{B}}/(\omega_{\mathrm{KI}}^2 - \omega^2)$ and $\kappa_{\mathrm{C}}/(\omega_{\mathrm{KI}}^2 - \omega^2)$ as the coefficient of the integrals in B and C, respectively, the results of the integrations in equation 6-9 can be obtained.

$$A_f = \kappa_{\mathrm{A}}\rho\left[\ln\left|\frac{\omega_{\mathrm{FI}}+\omega_f-\omega}{\omega_{\mathrm{BI}}+\omega_f-\omega}\right| + \ln\left|\frac{\omega_{\mathrm{FI}}+\omega_f+\omega}{\omega_{\mathrm{BI}}+\omega_f+\omega}\right|\right] \tag{12}$$

$$A_k = \kappa_{\mathrm{A}}\rho\left[\ln\left|\frac{\omega_{\mathrm{KF}}+\omega_k-\omega}{\omega_{\mathrm{KA}}+\omega_k-\omega}\right| + \ln\left|\frac{\omega_{\mathrm{KF}}+\omega_k+\omega}{\omega_{\mathrm{KA}}+\omega_k+\omega}\right|\right] \tag{13}$$

$$B = \frac{\kappa_{\mathrm{B}}\rho}{2}\left\{\frac{1}{\omega_{\mathrm{KI}}-\omega}\ln\left|\frac{\omega_{\mathrm{FI}}-\omega}{\omega_{\mathrm{BI}}-\omega}\right| + \frac{1}{\omega_{KI}+\omega}\ln\left|\frac{\omega_{\mathrm{FI}}+\omega}{\omega_{\mathrm{BI}}+\omega}\right|\right\} \tag{14}$$

$$C = \frac{\kappa_{\mathrm{C}}\rho}{2}\left\{\frac{1}{\omega_{\mathrm{KI}}-\omega}\ln\left|\frac{\omega_{\mathrm{KF}}-\omega}{\omega_{\mathrm{KA}}-\omega}\right| + \frac{1}{\omega_{\mathrm{KI}}+\omega}\ln\left|\frac{\omega_{\mathrm{KF}}+\omega}{\omega_{\mathrm{KA}}+\omega}\right|\right\} \tag{15}$$

Then, it can be inferred that the density of states near the Fermi level of the substrate may be in direct proportion to the polarizability tensor and thus contribute to the overall SERS enhancement.

Theoretical Calculation.

Density functional theory (DFT) simulations were performed within the generalized gradient approximation (GGA) using the VASP package, to study the optimize geometries and electronic properties of Al-intercalated tungsten oxide. The exchange-correlation energy was determined by the Perdew-Burke-Ernzerhof (PBE) function. A plane-wave energy cut-off of 450 eV and an energy convergence criterion of 10^{-5} eV for self-consistency were adopted. Supercells consisting of 9, 16, 25, 36, 49 primitive cubic WO_3 cells with an Al atom were employed to study the effect of fractional doping in the Al_xWO_3 system. The *k*-meshes were generated for different supercells with a *k*-point resolved value of 0.03. The insertion site of the Al atom was firstly investigated to be the face center of the WO_3 primitive cell, while the body center site was 2.9 eV higher in energy. Although the GW or hybrid functional calculations result in more accurate band gaps, the standard DFT calculations were performed in this study, since our main results are only based on the electron doping effects from the Al atoms rather than the band gaps of the WO_3.

Supplementary Notes

Supplementary Note 1. The physicochemical properties of the analyte molecules.

Rhodamine 6G (R6G) is a well-known laser dye, featured by its high photostability, high fluorescence quantum yield (ca. 0.95) and low cost. Structurally, R6G belongs to the family of xanthenes, which is featured by the bulky monoethylamino group with methyl as ortho-substituents in the xanthene skeleton, together with carboxylate ester (COOEt) group in the lateral phenyl ring (Supplementary Figure 8a). The carboxylate ester groups contribute toward the mobility of π-electron of the xanthene skeleton, resulting in the resonance structures of the xanthene moieties upon excitation. With the contributions from such resonance structures, the positive charge in the cationic R6G dye molecule can be stabilized across the 9-carbon atom in xanthene skeleton, showing good affinity to the negatively charged tungstic units existing in eletrochromic SERS substrates. Also, it is noted that the maximum absorption for R6G in aqueous solution is observed at ca. 530 nm (Supplementary Figure 8b), which exactly fits the used excitation wavelength (532.8 nm) during SERS measurements. Accordingly, when a R6G system is excited by 532 nm pulsed laser, it shows a molecular resonance Raman effect in addition to the normal SERS effect, which leads to larger SERS enhancements. Thus, the so-called surface-enhanced resonance Raman scattering (SERRS) effect could be recognized in our system, basing on the coincidence of incident photon energy and electronic transition in highly fluorescent R6G molecules.

Crystal violet or gentian violet (also known as methyl violet 10B or hexamethyl pararosaniline chloride) is a triarylmethane dye used as a histological stain and in Gram's method of classifying bacteria. When dissolved in water, the dye has a blue-violet color with an absorbance maximum at 590 nm and an extinction coefficient of 87,000 M^{-1} cm^{-1}.

Victoria Blue B is a widely used stain that was introduced to histology more than 100 years ago. It is also used in botany, bacteriology and cytology. When dissolved in water, the dye has a blue color with an absorbance maximum at 599 nm.

Supplementary Note 2. The morphology of the sputtered film in comparison with drop-casted film prepared from $W_{18}O_{49}$ nanowire suspension.

Morphologically, the sputtered tungsten oxide film before coloration shows a rather uniform surface, which is constructed by closely-packed coral-like structures in the size range of 200-400 nm (Figure 1a). After coloration, the surface structure of the films remains the same as the pristine film (Figure 1c). In contrast, the tungsten oxide film prepared by drop casting method shows the rough, loose and porous surface feature (Supplementary Figure 23).

Supplementary Note 3. The molecule fluorescence quenching effect on the electrochromic SERS substrate.

Rhodamine 6G (R6G) is known to be a highly fluorescent dye, featured by its high photostability, high fluorescence quantum yield (ca. 0.95) and low cost. It is observed that pure R6G dye exhibits a strong fluorescent emission at 551 nm in solution (Supplementary Figure 24a), which would prevent observation of the SERS spectrum. However, after the loading of R6G dye on the electrochromic substrate, the drastic quenching in the fluorescence intensity of R6G occurs with respect to the reference spectrum on SiO_2/Si substrate (Supplementary Figure 24b). The efficient fluorescence quenching for adsorbed analyte is probably ascribed to the metallic nature of the electrochromic substrates with high electron conductivity, which will facilitate the electron transfer or energy transfer to alleviate the charge-carrier recombination[10].

Supplementary Note 4. About the usefulness of colorimetric functionality.

As is well known, commercially available metallic SERS substrates based on well-organized hotspot structures often have the quality guarantee period emphasized in their user's manual. In fact, their performances such as activity and reproducibility will be gradually deteriorated once unpacked for use, since their delicate surface structuring is easily destroyed. Unfortunately, the performance degradation for metallic SERS substrates cannot be easily perceived by the naked eyes, because the

quality-degraded substrates have the same appearances as the fresh one, which may cause users great trouble in accurately analyzing of SERS signals. In contrast, the electrochromic SERS substrates with inherent colorimetric functionality have the ability to reveal their SERS activity through a color change observable by the naked eye, which provide a visual, rapid determination about the SERS-active status of SERS substrates.

Supplementary References

1. Grätzel, M. Photoelectrochemical cells. *Nature* **414**, 338-344 (2001).
2. May, M. M.; Lewerenz, H.-J.; Lackner, D.; Dimroth, F.; Hannappel, T. Efficient direct solar-to-hydrogen conversion by in situ interface transformation of a tandem structure. *Nat. Commun.* **6**, 8286 (2015).
3. Cai, W. B.; Ren, B.; Li, X. Q.; She, C. X.; Liu, F. M.; Cai, X. W.; Tian, Z. Q. Investigation of surface-enhanced Raman scattering from platinum electrodes using a confocal Raman microscope: dependence of surface roughening pretreatment. *Surf. Sci.* **406**, 9-22 (1998).
4. Jiang, L.; You, T.; Yin, P.; Shang, Y.; Zhang, D.; Guo, L.; Yang, S., Surface-enhanced Raman scattering spectra of adsorbates on Cu_2O nanospheres: charge-transfer and electromagnetic enhancement. *Nanoscale* **5** (7), 2784-2789 (2013).
5. Chen, J.; Shen, B.; Qin, G.; Hu, X.; Qian, L.; Wang, Z.; Li, S.; Ren, Y.; Zuo, L., Fabrication of Large-Area, High-Enhancement SERS Substrates with Tunable Interparticle Spacing and Application in Identifying Microorganisms at the Single Cell Level. *J. Phys. Chem. C* **116** (5), 3320-3328 (2012).
6. Cai, W. B.; Ren, B.; Li, X. Q.; She, C. X.; Liu, F. M.; Cai, X. W.; Tian, Z. Q., Investigation of surface-enhanced Raman scattering from platinum electrodes using a confocal Raman microscope: dependence of surface roughening pretreatment. *Surf. Sci.* **406** (1), 9-22 (1998).
7. Zhang, Q.; Li, X.; Yi, W.; Li, W.; Bai, H.; Liu, J.; Xi, G., Plasmonic MoO_2 Nanospheres as a Highly Sensitive and Stable Non-Noble Metal Substrate for

Multicomponent Surface-Enhanced Raman Analysis. *Anal. Chem.* **89** (21), 11765-11771(2017).

8. Lombardi, J. R.; Birke, R. L.; Lu, T. H.; Xu, J. Charge transfer theory of surface enhanced Raman spectroscopy: Herzberg-Teller contributions. *J. Chem. Phys.* **84**, 4174 (1986).
9. Albrecht, A. C. On the theory of Raman intensities. *J. Chem. Phys.* **34**, 1476 (1961).
10. Dulkeith, E. et al. Fluorescence quenching of dye molecules near gold nanoparticles: Radiative and nonradiative effects. *Phys. Rev. Lett.* **89**, 203002 (2002).
